# Draft Genome Sequence of the Fungus *Penicillium solitum* NJ1

**DOI:** 10.1128/genomeA.01176-16

**Published:** 2016-11-23

**Authors:** Guohua Yin, Yuliang Zhang, Kayla K. Pennerman, Sui Sheng T. Hua, Jiujiang Yu, Anping Guo, Zhixin Liu, Joan W. Bennett

**Affiliations:** a Key Laboratory of Biology and Genetic Resources of Tropical Crops, Ministry of Agriculture, Institute of Tropical Bioscience and Biotechnology, Chinese Academy of Tropical Agricultural Sciences, Haikou, Hainan, China; b Department of Plant Biology and Pathology, Rutgers, The State University of New Jersey, New Brunswick, New Jersey, USA; c U.S. Department of Agriculture, ARS, Western Regional Research Center, Albany, California, USA; d U.S. Department of Agriculture, ARS, Beltsville Agricultural Research Center, Beltsville, Maryland, USA

## Abstract

*Penicillium solitum* is one of the most prevalent species causing postharvest decay of pomaceous fruits during storage. Here, we report the draft genome of *P. solitum* strain NJ1, received as a transfer of a strain originally identified as *P. griseofulvum* by classical means.

## GENOME ANNOUNCEMENT

Many *Penicillium* species cause postharvest decay on apples and pears called “blue mold” (1). *P. solitum* is common on pomaceous fruits and is one of the most frequently found species of *Penicillium* (2). Although on apples it is a weak pathogen compared with *P. expansum* (2, 3), a recent study found that it was more aggressive on pear cultivars than on apple cultivars (4). A cosmopolitan species that is common in soils, it also has been isolated from sources as varied as marine sediments in Antarctica (5) and traditional sausages in Northern Greece (6). Furthermore, it produces solistatinol, a polyketide-derived compaction analogue (7). Compaction, also known as mevastatin, was the first drug discovered in the family of cholesterol-lowering compounds generically known as statins (8). In our previous research, we reported the draft genome of one *P. solitum* strain, RS1 (9). To further study the significance of *P. solitum* as a postharvest pathogen of pome fruits, as well as a producer of medically active compounds for the pharmaceutical industry, we report here the draft genome of a strain of *P. solitum*, designated NJ1.

This strain was received as a subculture of *Penicillium* sp. strain NRRL2159A, which originally had been isolated as a white sector from a colony of *P. griseofulvum*. When cultured in our laboratory, the isolate formed green-spored colonies. Furthermore, preliminary analysis of the ITS, BenA, and CaM loci indicated that it was *P. solitum*. Therefore, we renamed it as *P. solitum* strain NJ1.

Fresh spores of *P. solitum* NJ1 were inoculated in potato dextrose broth and incubated at 25°C for seven days, with shaking at 200 rpm. Total genomic DNA was extracted using an E.Z.N.A. Fungal DNA midi kit (OMEGA Bio-tek) according to the manufacturer’s instructions. Paired-end 450-bp, 2-kb, and 8-kb Illumina fragment reads were generated using an Illumina MiSeq benchtop sequencer, and the sequence depths reached 222×, 109×, and 120×, respectively. The genome was assembled using SOAPdenovo version 2.04 (http://soap.genomics.org.cn). The resulting assembly had 954 contigs, with an *N*_50_ value of 93,897 bp. The GC content of the genome was 47.95%. Based on the 17 *k*-mer statistical analysis, the estimated genome size of *P. solitum* NJ1 was about 32.5 Mb.

The genome sequence of *P. Solitum* NJ1 was annotated using the MAKER2 program (10). Preliminary annotation indicated that the genome contained 12,057 predicted genes, with an average length of 1,427 bp, comprising 54.2% of the whole genome. Repeated sequences totaled 365,865 bp, making up 1.15% of the genome. Our data will guide future molecular work with *P. solitum*, leading to a better understanding of this species as a postharvest pathogen and of its complex secondary metabolic capability.

### Accession number(s).

The genome sequence of *P. solitum* NJ1 has been deposited at DDBJ/ENA/GenBank under the accession number MJCB00000000. The version described in this paper is the first version, MJCB01000000.
